# Construction and identification of recombinant lentiviral vector containing HIV-1 Tat gene and its expression in 293T cells^☆^

**DOI:** 10.1016/S1674-8301(10)60009-7

**Published:** 2010-01

**Authors:** Bingbing Wei, Ninghan Feng, Feng Zhou, Chun Lu, Jiantang Su, Lixin Hua

**Affiliations:** a Department of Urology, the First Affiliated Hospital of Nanjing Medical University, Nanjing 210029, China; b Department of Microbiology and Immunology, Nanjing Medical University, Nanjing 210029, China

**Keywords:** prostate cancer, HIV-1, Tat

## Abstract

**Objective:**

To construct a lentiviral vector expressing HIV-1 Tat and identify its expression in 293T cells.

**Methods:**

The gene fragment of HIV-1 *Tat_101_* was subcloned to lentiviral transfer vector pHAGE-CMV-MCS-IZsGreen, which was named pHAGE-Tat. Then the constructed pHAGE-Tat was used to co-transfect the packing 293T cells, together with the packaging plasmids pMD2.G and psPAX2. The packaged viral particles designated LV-Tat were used to infect the 293T cells and the viral titer was calculated. The expression of HIV-1 Tat in 293T cells was confirmed using RT-PCR and western blot.

**Results:**

The recombinant lentiviral vector was successfully constructed and could express HIV-1 Tat in 293T cells. The virus titer was 5.73×10^6^ ifu/ml.

**Conclusion:**

The successfully constructed recombinant lentiviral vector makes a strong foundation for further exploring the possible role of HIV-1 Tat in the development of prostate cancer.

## INTRODUCTION

Prostate cancer is one of the most common malignant diseases in men[1]. The mechanism of prostatic carcinogenesis and development is still unclear. As is known, the oncoviruses that predispose infected individuals to be more vulnerable to cancer include Kaposi's sarcoma-associated herpesvirus (KSHV)[2]–[4], Epstein–Barr virus[5],[6], and human papilloma virus[7]–[9], human T-lymphotropic virus[10],[11] and hepatitis virus[12]–[14]. As a result, research on the association of oncoviruses with carcinogenesis has become a focus for researchers all over the world.

KSHV, also known as herpesvirus-8 (HHV-8), is a gamma-2 herpesvirus and is B-lymphotropic, similar to Epstein-Barr virus[15]. It is the primary causative agent of Kaposi's sarcoma (KS)[16]–[19], as well as a rare lymphoma (primary effusion lymphoma)[20]. Because of the increasing number of human beings infected with KSHV and HIV-1, it has become one of the most commonly studied oncoviruses and has attracted our attention. It was reported that HHV-8 was present in the male reproductive tract and can be transmitted through sexual contact[21],[22]. A recent study found an increased prevalence of antibodies against HHV-8 among men with prostate cancer, when compared with control subjects[22]. An Italian group reported a high incidence of detection of HHV-8 sequences in prostatic tissue from persons without AIDS or KS[23]. In the Afro-Caribbean population on the island of Tobago, HHV-8 seroprevalence was significantly higher among men with biopsy-proven prostate cancer than among age-matched control subjects who had normal levels of prostate-specific antigen (PSA) and normal digital rectal examinations. Hayes *et al*[24] has reported an elevated risk of prostate cancer among men with sexually transmitted diseases. Because HHV-8 has oncogenic potential and both HHV-8 and HIV-1 act as sexually transmitted agents, carcinogenesis and development of prostate cancer should be associated with the biochemical effects of either or both KSHV and HIV-1.

The transactivative transcription protein (Tat), a HIV-1-encoded protein that promotes replication, can be released by HIV-1-infected cells to the extracellular space. One study indicated that HIV-1 Tat could participate in KS pathogenesis by inducing KSHV replication and increasing KSHV viral load[25]. So HIV-1 Tat could directly or indirectly affect the carcinogenesis and development of prostate cancer. In an attempt to better understand the role of HIV-1 Tat, we construct a lentiviral vector expressing HIV-1 Tat, which could be used to infect prostate cancer lines *in vitro* and maintain long-term expression of HIV-1 Tat in lentivirus-infected prostate cancer lines, thus providing a foundation for the further research.

## MATERIALS AND METHODS

### Materials

The lentiviral packaging system consisting of pHAGE-CMV-MCS-IZsGreen, pMD2.G and psPAX2 was a gift kindly supplied by Dr. Huang Zan (Ben May Institute for Cancer Research, The University of Chicago, Chicago, USA). HEK293T packaging cells (kindly provided by Professor Lu Chun, Department of Microbiology and Immunology, Nanjing Medical University) were stored in our lab and cultured in DMEM medium plus 10%FBS (Gibco BRL, USA), 2 mmol/L L-glutamine, 100 U/ml penicillin, and 100 µg/ml streptomycin at 37°C in a humidified 5% CO_2_ atmosphere. *Escherichia coli* *(E.coli)* DH5α was purchased from Nanjing Tianwei Corporation. pEV plasmid, also named pcDNA3.1+/Tat_101_ 2exflag contained an 86-amino acid full length Tat natural sequence, adding 15-amino acids and finally adhering to DYKDDDDK sequence which builds up Flag was kindly provided by Dr. Rice (Southwest Medical Center, Texas University, USA). DNA gel extraction kit and Lipofectamine^TM^ 2000 were respectively obtained from Promega, USA and Invitrogen, USA. TRIzol reagent was purchased from Invitrogen and reverse transcription reagents were obtained from Applied Biosystems (Foster City, CA). All the restriction enzymes and markers (Lambda DNA/HindIII+EcoRI) were purchased from Fermentas MBI, USA. Primers were synthesized by Shanghai Shennengbocai Company. Monoclonal antibodies, anti-Flag M2 and anti-β-actin, were obtained from Sigma Company, USA, and ECL western blot detection regent kits were purchased from Amersham-Pharmacia, USA.

### Construction of Tat-expressed transfer vector pHAGE-Tat

The gene fragment of HIV-1 Tat was amplified from template plasmid pEV using the designed primers below:

Upper Primer-*Tat*: 5′-CGT AGC TAG CGC CAC CAT GGA GCC AGT AG-3′

Lower Primer-*Tat*: 5′-GCA CGG ATC CCT ACT TGT CAT CGT CGT CCT TG-3′

The introduced restriction sites, BamHI and NheI, are underlined, respectively. The cycle parameters were as follows: 95°C for 5 min, then 35 cycles of 94°C 1 min, 63°C for 30s, 72°C 1 min, followed by a final extension of 72°C for 5 min. The PCR product was around 360bp in length.

The purified PCR product and transfer vector were respectively digested with the two enzymes, BamHI and NheI, for 3 h. The enzyme-digested product was purified again with the gel extraction kit. They were ligated using T4 DNA ligase at 20°C overnight under conditions that permitted the existence of the corresponding adhesive ends. The ligated product was used to transfect *E. coli* DH5α. Then the growing positive colonies were picked out and gently mixed with LB broth containing AMP, and the corresponding plasmids were extracted using the plasmid extraction kit. Finally, the insert was confirmed by PCR as designed above, enzyme digestion and nucleotide sequence analysis.

### Production of Tat-expressed recombinant lentivirus particles (LV-Tat)

The constructed Tat-containing transfer vector pHAGE-Tat was co-transfected into the 293T cells using lipofectamine^TM^ 2000, together with the other two plasmids, pMD2.G and psPAX2. Forty-eight hours later, the supernatant was collected and the fresh medium was added. Seventy-two hours later, the supernatant was collected again. The collected supernatant was gently mixed, then centrifuged at 4,000g, at 4°C for 5 min. The supernatant was stored at -70°C for use in subsequent experiments.

### Measurement of the viral titer of recombinant lentiviral vector

The 293T cells were cultured in DMEM supplemented with 10% FBS and seeded onto 24-well plates. The concentration of virus collected above acted as stock and this viral stock was serially diluted with DMEM(10^−1^,10^−2^,10^−3^,10^−4^,10^−5^,10^−6^). Each dilution was used to infect 293T cells. The infections were conducted in triplicate and corresponding negative controls to which no virus were added were also performed. Forty-eight hours later, the expression of IZsGreen was detected using fluorescence microscopy. Fluorescing cells were counted. The well in which the average number of fluorescing cells was between 10 and 100 was used to evaluate the virus titer. The virus titer was obtained by calculating infectious units (ifu)/ml for each well as follows: [(infected cells/field)×(fields/well)]/[volume of virus (ml)×(dilution factor)]. The 293T cells were seeded onto 6-well plates at 5×10^5^/well, and the above recombinant lentivirus particles were added 48 hours later, Flow cytometry (FCM) was used to detect the efficiency of infection using standard methods.

## RESULTS

### Construction of lentiviral vector containing HIV-1 *Tat*

**Fig. 1:**
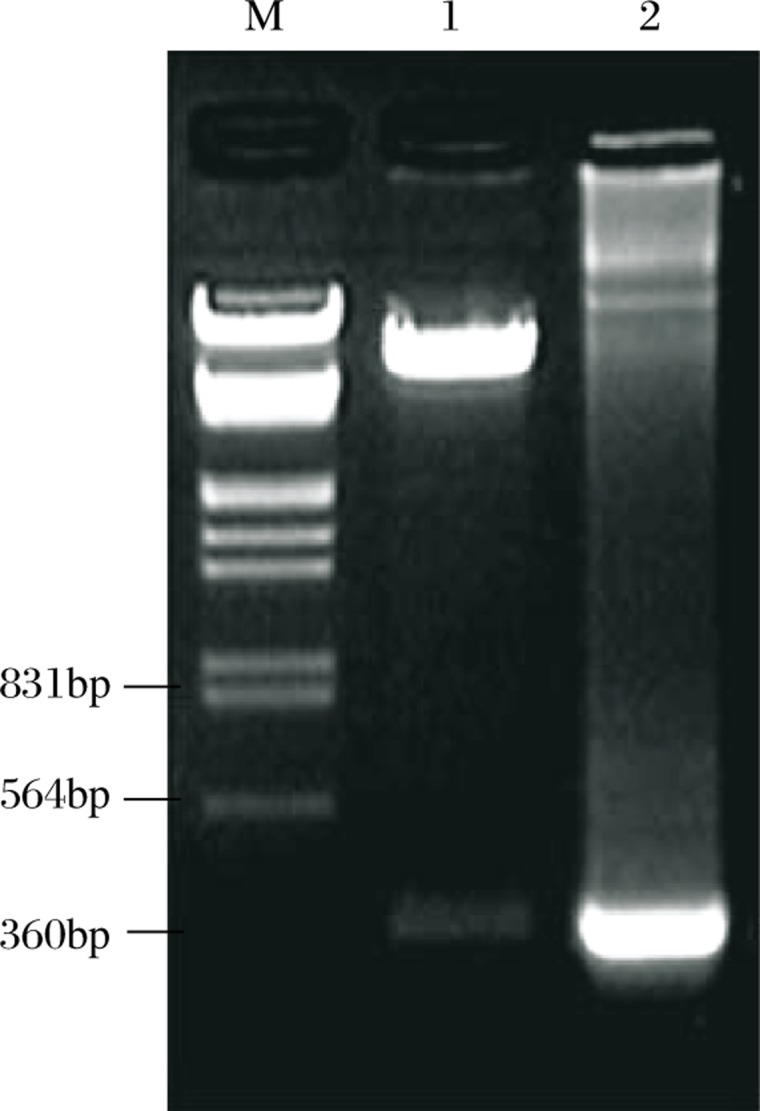
The identification of Tat-expressed transfer vector pHAGE-Tat. M: DNA marker; Lane 1: Restriction enzyme digestion analysis of recombinant plasmid pHAGE-Tat; Lane 2: PCR-analysis of recombinant plasmid pHAGE-Tat.

**Fig. 2:**
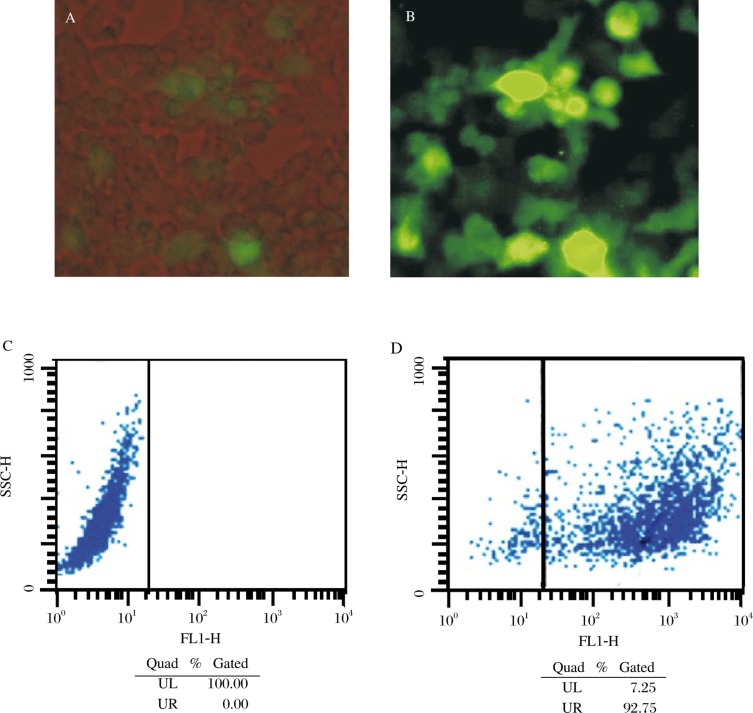
Detection of ILS Green expressed in 293T cell by light microscope (A: ×100, 72 h) flurescence microscope (B: ×100, 72 h) and FCM (C: control group without infection of LV-Tat; D: experimental group infected with LV-Tat, detected on 48 h after infection).

The gene fragment of HIV-1 *Tat* was amplified from template plasmid and then subcoloned into the MCS of lentiviral transfer vector pHAGE. The insert of HIV-1 *Tat* was confirmed by PCR, double-enzyme digestion (***Fig. 1***) and gene sequencing (data not shown). pHAGE-Tat, pMD2.G and psPAX2 were used to co-transfect the 293T cells, and the viral particles were collected 48 and 72 hours later. At the same time, the IZsGreen expressed by 293T cells was detected using fluorescence microscopy (***Fig. 2A*** ***and*** ***2B***).

**Fig. 3:**
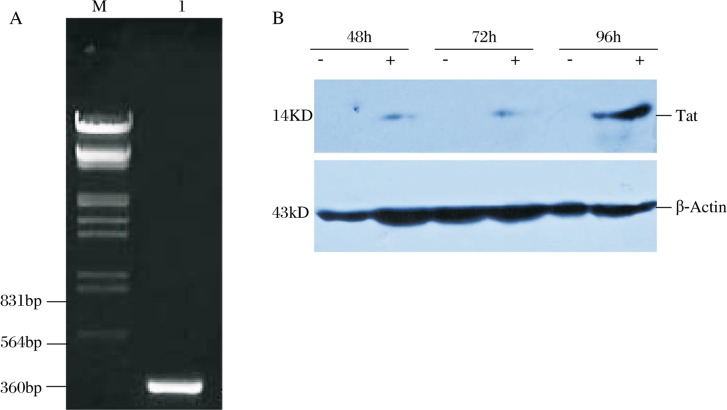
Expression of HLV-Tat in 293T cell detected by RT-PCR and wester blot. A: mRNA expression of HLV-Tat (lane l; M:Marker). B: Protein expression of HLV-Tat in 293T cell infected with LV-pHAGE (negative control) and LV-Tat for 48h, 72h and 96h respectively.

### Titer measurement of lentivirus

The 293T cells were seeded onto 24-well plates at 3×10^4^/well, and the packaged lentivirus particles were used to infect them. Forty-eight hours later, fluorescing cells were counted using fluorescence microscopy. The calculated viral titer was 5.73×10^6^ ifu/ml. When 1ml of the lentivirus particles was added, the efficiency of infection was around 90% as detected by FCM (***Fig. 2C*** ***and*** ***2D***).

### The detection of HIV-1 Tat in 293T cells using RT-PCR.

The packaged lentivirus particles were used to infect the 293T cells, and 48 hours later the RNA was isolated, from which cDNA was synthesized. PCR was then used to detect the transcription of HIV-1 *Tat* in 293T cells (***Fig. 3A***).

### The expression of HIV-1 Tat in 293T cells using western blot.

The 293T cells were infected with LV-pHAGE (negative controls did not express HIV-1 *Tat*) and LV-Tat. Whole-cell extracts of protein isolated from 293T cells infected with LV-pHAGE and LV-Tat for 48, 72 and 96h were detected using western blot (***Fig. 3B***). The constructed recombinant lentiviral vector containing HIV-1 *Tat* could express Tat in 293T cells.

## DISCUSSION

The theory that viruses could lead to cancer began to be developed in 1911 as proposed by Peyton Rous. By the early 1950s, it was observed that viruses could remove and incorporate genes and genetic materials in different cells, and these became known as oncoviruses. Many of these oncoviruses have been found to cause cancer, including Epstein-Barr virus [5]–[6], Kaposi's sarcoma-associated herpesvirus[2]–[4], human papillomavirus[7]–[9], human T-lymphotropic virus[10],[11] and hepatitis virus[12]–[14]. It was reported that these new viral genes inserted into host cells could make the cells cancerous.

HHV-8 has oncogenic potential and is the primary causative agent of KS, as well as a rare lymphoma (primary effusion lymphoma). While there is accumulating evidence that HHV-8 may play a role in the development of prostate cancer, whether there is an unequivocal cause and effective association between HHV-8 and prostate cancer has yet to be established. Additionally, it has been demonstrated that the HIV-1 *Tat* gene product can affect the replication cycle of HHV-8. We speculate that HIV-1 Tat indirectly influences the development of prostate cancer by affecting the lytic replication of HHV-8[25]. The first step in our attempt to demonstrate the validity of our speculation was to construct a lentiviral vector that could make the infected cells express HIV-1 Tat.

Conventional methods for transgenesis maintain relatively low transgenic efficiency, and this has opened the door for alternative approaches, including the use of lentiviral vectors. Lentiviral vectors are an appealing tool for transgenesis partly because of their capability to incorporate into genomic DNA with high efficiency, especially in cells that are not actively dividing. Lentiviral vector-mediated transgenic expression can also be maintained for long periods of time. Recent studies documented high efficiencies for lentiviral transgenesis, even in various animal species and strains. These advantages broadened its application as an appealing tool for transgenesis. Hence, we specifically chose a lentiviral vector for this transgenesis study. We found that the HIV-1 Tat could be isolated from 293T cells infected with LV-pHAGE and LV-Tat for 48, 72 and 96h. However, the protein was not detected for 24h. On the whole, the constructed recombinant lentiviral vector containing HIV-1 *Tat* works well in 293T cells.

In this study, the recombinant lentiviral vectors expressing HIV-1 Tat were successfully constructed and viral particles with a high titer were also packaged. This methodology provides the basis for our further research.
